# Prognostic Implications of Immune-Related Genes’ (IRGs) Signature Models in Cervical Cancer and Endometrial Cancer

**DOI:** 10.3389/fgene.2020.00725

**Published:** 2020-07-21

**Authors:** Hao Ding, Guan-Lan Fan, Yue-Xiong Yi, Wei Zhang, Xiao-Xing Xiong, Omer Kamal Mahgoub

**Affiliations:** ^1^Department of Gynecology, Zhongnan Hospital of Wuhan University, Wuhan, China; ^2^Central Laboratory, Renmin Hospital of Wuhan University, Wuhan, China

**Keywords:** immune-related genes, cervical cancer, endometrial cancer, TCGA, prognostic model

## Abstract

Cervical cancer and endometrial cancer remain serious threats to women’s health. Even though some patients can be treated with surgery plus chemoradiotherapy as a conventional option, the overall efficacy is deemed unsatisfactory. As such, the development for new treatment approaches is truly necessary. In recent years, immunotherapy has been widely used in clinical practice and it is an area of great interest that researchers are keeping attention on. However, a thorough immune-related genes (IRGs) study for cervical cancer and endometrial cancer is still lacking. We therefore aim to make a comprehensive evaluation of IRGs through bioinformatics and large databases, and also investigate the relationship between the two types of cancer. We reviewed the transcriptome RNAs of IRGs and clinical data based on the TCGA database. Survival-associated IRGs in cervical/endometrial cancer were identified using univariable and multivariable Cox proportional-hazard regression analysis for developing an IRG signature model to evaluate the risk of patients. In the end, this model was validated based on the enrichment analyses through GO, KEGG, and GSEA pathways, Kaplan-Meier survival curve, ROC curves, and immune cell infiltration. Our results showed that out of 25/23 survival-associated IRGs for cervical/endometrial cancer, 13/12 warranted further examination by multivariate Cox proportional-hazard regression analysis and were selected to develop an IRGs signature model. As a result, enrichment analyses for high-risk groups indicated main enriched pathways were associated with tumor development and progression, and statistical differences were found between high-risk and low-risk groups as shown by Kaplan-Meier survival curve. This model could be used as an independent measure for risk assessment and was considered relevant to immune cell infiltration, but it had nothing to do with clinicopathological characteristics. In summary, based on comprehensive analysis, we obtained the IRGs signature model in cervical cancer (*LTA, TFRC, TYK2, DLL4, CSK, JUND, NFATC4, SBDS, FLT1, IL17RD, IL3RA, SDC1, PLAU*) and endometrial cancer (*LTA, PSMC4, KAL1, TNF, SBDS, HDGF, LTB, HTR3E, NR2F1, NR3C1, PGR, CBLC*), which can effectively evaluate the prognosis and risk of patients and provide justification in immunology for further researches.

## Introduction

Cervical cancer is the fourth most commonly occurring cancer in women worldwide (Yang et al., 2019), for which a major cause is chronic infection with high-risk HPV types (HPV types 16 and 18) (Cohen et al., 2019). This condition is considered the leading cause of death and disability for women, although progress has been made for diagnostic methods and treatment in recent years in the context of improved test panels that provide detailed screening around the world. In 2018, approximately 570,000 patients were diagnosed with cervical cancer and 31,000 died from it globally (Bray et al., 2018). In Japan, it has been estimated that there are 13,000 new cases and 3,500 deaths associated with cervical cancer each year (Ishikawa et al., 2020). The 5-year survival rate can be encouraging for local cervical cancer, as approximately 75–85% after effective treatments such as surgery. However, the 5-year survival rate for recurrence is approximately 15% (Liu et al., 2019b). Histopathologically, squamous cell carcinoma accounts for about 80–85% and adenocarcinoma about 15–20% (Yang et al., 2019). Traditionally, a patient may be treated with surgical removal of the lesions and adjacent lymph nodes in combination with cycles of radiotherapy and chemotherapy (Cosper et al., 2019). Recently, immunotherapy has been increasingly used in clinical settings (Crusz and Miller, 2020) and has now becomes one of the important areas of cancer research.

Endometrial cancer is another very common gynecological tumor, ranking as the sixth cause of cancer incidence in women following breast cancer, colorectal cancer, lung cancer, cervical cancer, and thyroid cancer (Bray et al., 2018). Statistics show that the incidence of endometrial cancer is second only to cervical cancer (Feng et al., 2019; Zhou and Ling, 2019) among gynecological malignancies in China. The survival rate for endometrial cancer varies with tumor progression; there was a big difference in 5-year survival rate by 83–97% in localized to 43–67% in stage III, and finally only 13–25% in stage IV (Liu, 2019). Traditional treatment options including surgery, radiotherapy, and chemotherapy can be effective for the condition in early stages but advanced diseases are not significantly responsive (Miller et al., 2020). As novel immunotherapies are being used to treat endometrial cancer (Grywalska et al., 2019), a new option is now available for doctors (Lynam et al., 2019).

Immunotherapies for cancer have attracted more and more attention from scientific researchers (Irvine and Dane, 2020). In recent years, traditional modalities have found more limitations to the treatment of cancer. Immunotherapies have provided more opportunities to modern precision medicine and personalized medicine (Martin et al., 2020). In fact, many immunotherapeutic methods have been applied in clinical practice, such as the typical immune checkpoint inhibitor that targets programmed cell death protein 1 (PD-1) for lung cancer (Gainor et al., 2020) and breast cancer (Barroso-Sousa et al., 2020), as well as CD19-specific CART (Shen et al., 2019) immune cell therapy for leukemia (June et al., 2018). In addition, immunotherapies are also widely used to treat gynecological tumors (Rubinstein and Makker, 2020). Existing studies on immunotherapy for cervical cancer focus mainly on human papillomavirus vaccine, immune checkpoint inhibitors, and adoptive cellular therapy. The main biological mechanism of the human papillomavirus vaccine is the viral vectors expressing HPV-16 or -18 (E6 or E7) to stimulate the body’s immune response to malignant cells. These vaccines can be divided into two categories – prophylactic and therapeutic. Currently, there are three clinically available prophylactic human papillomavirus vaccines – Gardasil, Cervarix, and Gardasil 9 – that were approved by the U.S. Food and Drug Administration (FDA) in 2006, 2009, and 2016, respectively (Matanes and Gotlieb, 2019). Therapeutic human papillomavirus vaccine is also an important part of vaccine research, including live vector, nucleic acid, protein, whole cell, and combinatorial vaccines. However, although there are some promising vaccine candidates (Vici et al., 2016; Yang et al., 2016; Kim, 2017), there are currently no vaccine products available for human use. For immune checkpoint inhibitors, anti-programmed death 1 (PD-1) and anti-programmed death ligand 1 (PD-L1) immunoglobulin, as an important representative, have been the focus of research, and many drugs, such as pembrolizumab and nivolumab, have achieved encouraging results and were approved by the FDA (Wang and Li, 2019). At present, studies on adoptive cellular therapy in cervical cancer are insufficient. While some scholars have confirmed the efficacy of human papillomavirus-targeted tumor-infiltrating T lymphocytes (TILs) in cervical cancer (Stevanovic et al., 2015), they still face many problems. The main challenge lies in how to effectively identify the tumor-associated antigens (TAAs) from individual patients and how to amplify the TILs while inducing a targeted immune response to these tumor sites, which became the focus in subsequent studies. Compared to immunological studies on cervical cancer, studies on endometrial cancer are relatively few and mainly focus on the immune checkpoint inhibitors. Studies have shown that PD-1 and PD-L1 are expressed in 80% of primary endometrial carcinoma patients and almost 100% of metastatic tumors (Mo et al., 2016), The inhibitor pembrolizumab was FDA-approved for use in microsatellite instability-high (MSI-H) or mismatch repair (MMR)-deficient endometrial carcinoma patients (Le et al., 2017). Studies (Maskey et al., 2019) have shown that the number of CD4+ and CD8+ lymphocytes is not similar between normal cervical cells infected by HPV and cervical cancer cells, and this difference becomes more complicated for epithelial and stromal layers in cervical tissues. Based on a study of endometrial cancer, it was (Zhou and Ling, 2019) found that the survival rate correlated with the number of cytotoxic T lymphocytes. Despite the fact that *in vivo* and *in vitro* experiments are performed during plenty of studies on immune cell changes in gynecologic tumors, a more comprehensive and specific immune mechanism is still unclear.

As modern high-throughput sequencing technology is being improved and rapid growth is achieved in computer science (Ma et al., 2019), more and more free of charge, large-scale, and comprehensive gene transcriptomics as well as relevant clinical databases are available, which makes it possible to provide comprehensive analyses of genetic molecular biomarkers in a more accurate and fast fashion. These molecular biomarkers play an important role in predicting the prognosis of patients and evaluating their risk levels. Therefore, we hope to further explore those data that provide details in immune related genes (IRGs) for patients with cervical cancer and those with endometrial cancer. Beyond that, efforts will also be made to evaluate and predict the prognosis of patients using these molecular biomarkers or other gene signatures. By combining the gene expression profiles and clinical data of IRGs with bioinformatics statistical methods, we obtained and analyzed those IRGs signatures and then verified them in patients with cervical cancer and those with endometrial cancer. These results will provide us a basic idea for follow-up and in-depth studies on these IRGs, thus laying foundation for precise and individualized medical treatment.

## Materials and Methods

### Clinical Samples and Data Acquisition

For cervical and endometrial cancers, transcriptome RNA-sequencing data from FPKM file as well as clinical data were downloaded from The Cancer Genome Atlas (TCGA) database containing 3 non-tumor samples and 304 tumor samples from patients with cervical cancer, and 35 non-tumor samples and 543 tumor samples from those with endometrial cancer. All clinical data and transcriptome data did not correspond exactly because the clinical data were not completely provided, leading to exclusion from the subsequent analyses. Immune-related genes (IRGs) were derived from the Immunology Database and Analysis Portal (ImmPort) system (Bhattacharya et al., 2014) which was continuously updated and maintained to provide immune-related data that had endorsement by scholars. These resulting genes were thought to be involved in human’s immune-related activities.

### Differential Gene Analysis and Enrichment Analysis

All of these genes, including immune-related genes (IRGs) and all transcriptome RNA-sequencing genes that were differentially expressed in normal and tumor samples, were screened in association with cervical and endometrial cancer, respectively, through R-Limma package (R version 3.6.1), and the screening criteria were met based on false discovery rate (FDR) < 0.05 and log2 |fold change| > 1. Functional enrichment analyses through GO and KEGG pathways were conducted for differentially expressed IRGs using the online database webgestalt (Liao et al., 2019)^1^.

### Identification of Survival-Associated IRGs

We extracted the clinical data of overall survival (OS) time and survival state corresponding to cervical cancer and endometrial cancer, respectively, and the transcriptome of IRGs combined with corresponding clinical data to perform survival analysis and thus identify survival-associated IRGs using univariate Cox proportional hazard regression. To meet the screening criteria, *p* < 0.05 and *p* < 0.01 were defined for cervical cancer and endometrial cancer, respectively. Since many different IRGs were found for endometrial cancer, which was not helpful for subsequent analyses, more appropriate screening criteria should be followed.

### Screening of Transcription Factors (TFs) and Construction of Networks

Three hundred and eighteen transcription factors (TFs) were downloaded from the cistrome online database^2^ to figure out the differential genes in cervical and endometrial cancer, respectively, in a similar way used for IRG selection, using R-limma package (R version 3.6.1). The selection criteria were defined as false discovery rate (FDR) < 0.05 and log2 |fold change| > 1. Subsequently, the differentially expressed TFs and selected survival-associated IRGs were used to establish regulatory networks by Pearson correlation analysis with correlation coefficient > 0.4 at *p* < 0.001 after which the regulatory networks were imported into the cytospace software (version 3.7.2) for visual procedures.

### Establishment and Evaluation of the IRG Signature Model

The survival-associated IRGs were further screened to establish the IRGs signature model, which was examined by multivariate Cox proportional-hazard regression analysis. This model would be used for subsequent evaluation and analysis of risk measures for the patients’ risk values after assigning these patients into high-risk and low-risk groups. The risk score for each patient was computed using the formula as follows:

$$\text{risk score}=\sum_{\text{k}=1}^{\text{n}}\text{Coe}\;{\text{f}}_{k}^{*}{\text{X}}_{\text{k}}$$

where Coef_k_ represents the coefficient and X_k_ represents the expression level of each IRG. Subsequently, the validity of the IRGs signature model was evaluated by analyzing the difference between high- and low-risk groups using the Kaplan-Meier survival curve, Receiver Operating Characteristic (ROC) curve and heatmap. Similarly, Gene Set Enrichment Analysis (GSEA) was applied to compare signaling pathways and biological processes between high and low risk groups by GSEA (version 4.0.3) software. The landscape of genetic alterations across these IRGs in the signature model was examined through the online database cbioportal^3^.

### Evaluation of IRGs’ Signature Model Along With Clinicopathological Characteristics and Tumor-Infiltrating Immune Cells

Whether the patient risk score could be used as an independent prognostic measure was further evaluated by univariate and multivariate Cox proportional-hazard regression analyses. The tumor infiltrating immune cell index were download from the online Tumor IMmune Estimation Resource (TIMER) (Li et al., 2017)^4^, which provided detailed information about infiltrating immune cells including B cells, T cells, macrophages, neutrophils, and dendritic cells. Acceptable compatibility between these data and TCGA database is maintained; that is why the information thereof has been widely used in scientific researches in recent years. Therefore, it is helpful for us to further understand the changes of immune cells in tumor tissues. The relevance between risk scores and infiltrating immune cells was investigated herein using Pearson correlation analysis.

### Statistical Analysis

All data were processed using the R software (version 3.6.1). The independent samples *t*-test was used to evaluate the relationship between risk scores and clinicopathological characteristics, and *P* < 0.05 was considered statistically significant. For Kaplan-Meier survival curves, the log-rank test was performed to demonstrate if there could be significant difference in OS between groups. Univariate and multivariate Cox proportional-hazard regression analyses were used to access the association between risk scores and OS. The area under the ROC curve (AUC) was measured for indicating the accuracy of prognosis as shown by the IRG signature model. All these analyses were performed at a significance level of *P* < 0.05.

## Results

### Identification of Differentially Expressed IRGs

**FIGURE 1:**
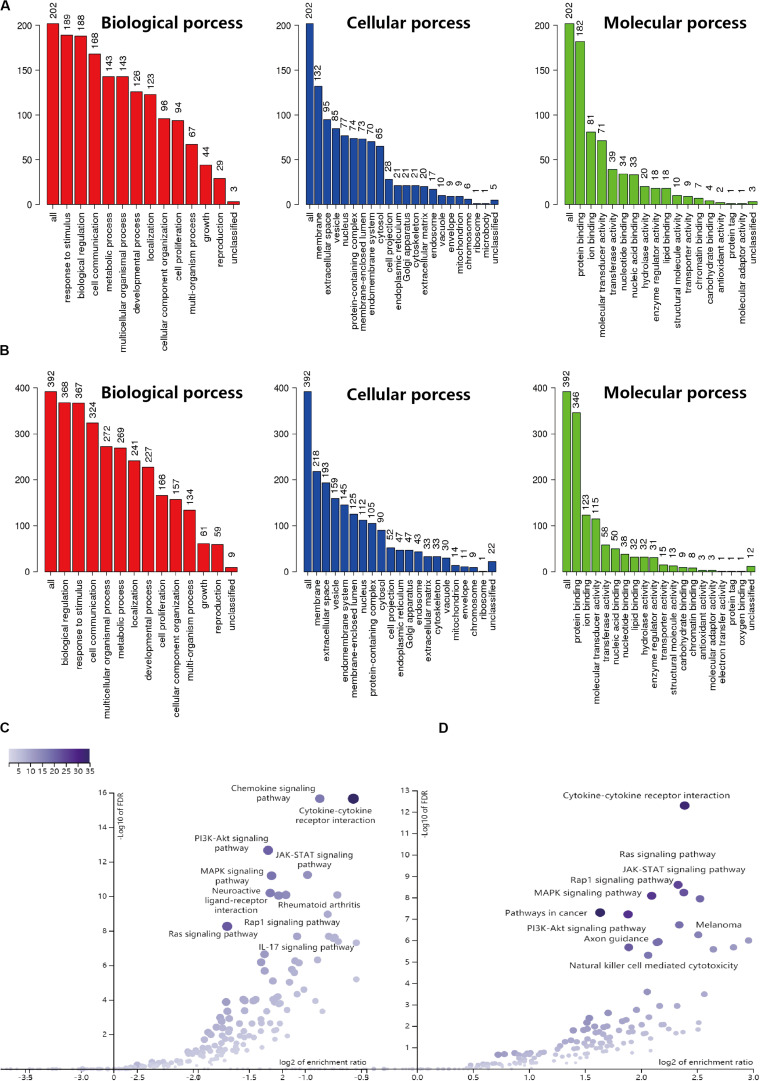
GO and KEGG enrichment result for IRGs in CESC and UCEC. **(A)** GO enrichment analysis for CESC, the vertical axis represents the number of differentially expressed IRGs. **(B)** GO enrichment analysis for UCEC, the vertical axis represents the number of differentially expressed IRGs. **(C)** Volcano of KEGG enrichment result for CESC. **(D)** Volcano of KEGG enrichment result for UCEC.

Based on the results derived from the R software, we found that there were 3192 differentially expressed genes in cervical cancer, including 1833 upregulated and 1359 downregulated; and 5665 differentially expressed genes in endometrial cancer, including 3316 upregulated and 2349 downregulated. A total of 2498 immune-related genes (IRGs) are described in the Immunology Database and Analysis Portal (ImmPort). We extracted differentially-expressed IRGs common to the TCGA and the ImmPort, which yielded 88 upregulated and 117 downregulated for cervical cancer, along with 226 upregulated and 171 downregulated for endometrial cancer. During enrichment analyses for these differentially expressed IRGs, the cervix-related genes were mainly found to be involved in “response to stimulus,” “biological regulation,” and “cell communication” for GO enrichment, and “cytokine-cytokine receptor interaction,” “Ras signaling pathway,” and “MAPK signaling pathway” as shown in Kyoto Encyclopedia of Genes and Genomes (KEGG). In comparison, endometrial cancer related genes showed biological processes in a similar manner, as they were also mainly involved in “biological regulation,” “response to stimulus,” and “cell communication” for GO enrichment, and “cytokine-cytokine receptor interaction,” “chemokine signaling pathway,” and “PI3K-Akt signaling pathway” as shown in Kyoto Encyclopedia of Genes and Genomes (KEGG) (Figure 1). The above findings suggested that these IRGs were strongly associated with the development, progression and invasion of tumors.

### Identification of Survival-Associated IRGs

**FIGURE 2:**
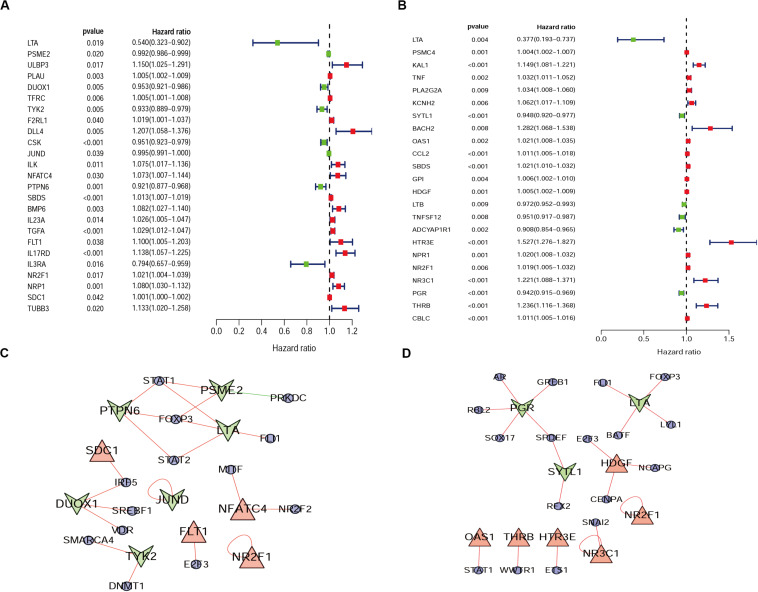
Forest plot of survival-associated IRGs and IRGs-TFs regulatory network. **(A)** Survival-associated IRGs in cervical cancer. **(B)** Survival-associated IRGs in endometrial cancer, red dots represent high-risk genes (HR > 1), and green dots represent low-risk genes (HR < 1). The IRGs-TFs regulatory network in cervical cancer **(C)** and endometrial cancer **(D)**. The blue ellipse represent TFs, the triangle and taper represent IRGs, red/green represent high-Risk IRGs/low-Risk IRGs, red/green lines represent TFs positive/negative regulation IRGs.

From the previous step, we obtained the differentially expressed IRGs. However, in our clinical studies, we paid more attention to the IRGs that were associated with the survival and prognosis of patients because these genes may be the key biomarkers for evaluating patients. During further screening, we obtained 25 survival-associated IRGs for cervical cancer and 23 for endometrial cancer, respectively (Figures 2A,B).

### Identification of Differentially Expressed TFs and Construction of IRGs-TFs Regulatory Network

**FIGURE 3:**
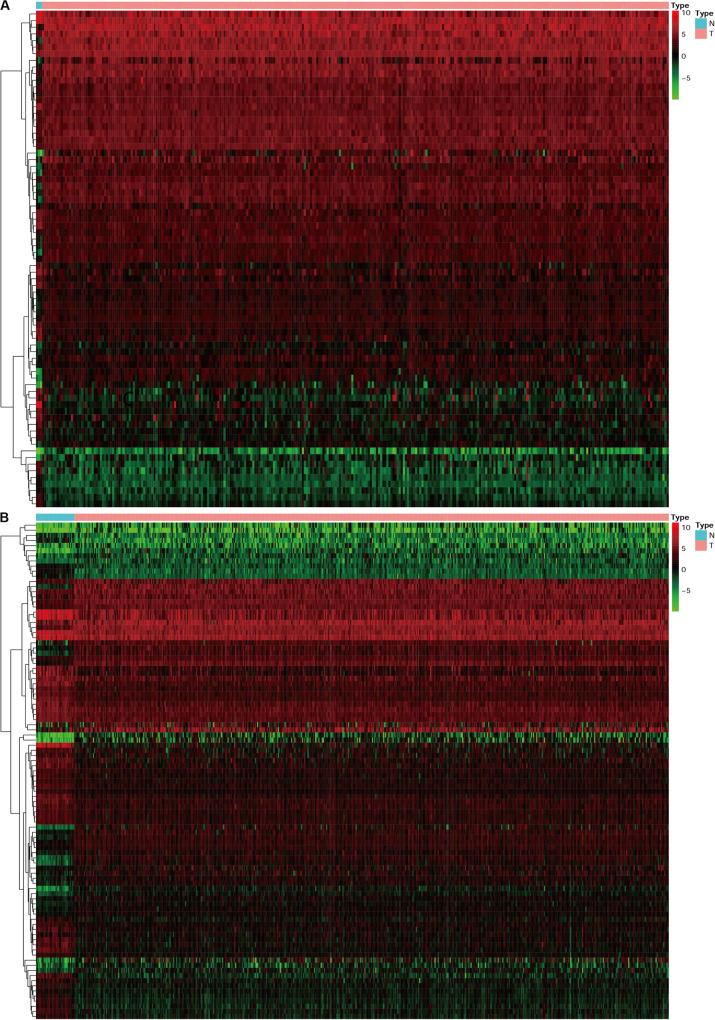
The heatmap for differentially expressed TFs in cervical cancer and endometrial cancer. N represent normal, T represent tumor or cancer. **(A)** Cervical cancer. **(B)** Endometrial cancer.

Progress has been made for researches on the change of DNA transcription factors (TFs) level in tumors, which is always the important direction for biological processes. By establishing the matrix for these TFs corresponding to the gene expression profiles, we found that there were 47 upregulated and 28 downregulated TFs in cervical cancer, as well as 44 upregulated and 53 downregulated TFs in endometrial cancer (Figure 3). The gene expression profiles were then extracted for survival-associated IRGs and differential expression TFs, respectively, to construct the IRGs-TFs regulatory network. As shown in the network diagram, the network for cervical cancer tissues was formed by 13 TFs and 10 IRGs. Similarly, 17 TFs and 9 IRGs formed the regulatory network for endometrial cancer. We found the IRG LTA in the regulatory network for both cervical cancer and endometrial cancer. As shown in the regulatory network, TFs STAT1 and FOXP3 were involved in regulatory relationship with multiple IRGs for cervical cancer, while IRGs *PGR* and *LTA* were associated with multiple TFs for endometrial cancer (Figures 2C,D). Both STAT1 and STAT2 are important members of the family of signal transducer and activator of transcription (STAT), but STAT1 plays the more important role (Verhoeven et al., 2020). Current studies have proven that STAT1 is an important activating mediator of type I and type II interferon (IFN), participating in the body’s immune defense response against foreign pathogens and other viruses (Zhang et al., 2017). The biological function of STAT1 is still controversial and unclear. Studies of breast (Hou et al., 2018) and ovarian cancer (Tian et al., 2018) found that STAT1 is overexpressed in malignant tumors and plays an oncogenic role. However, studies on colorectal cancer (Crncec et al., 2018) and other breast cancers (Varikuti et al., 2017) found that TAT1 may act as a tumor suppressor. Even a recent meta-analysis of TAT1 in multiple types of tumors reported that the prognostic factor of STAT1 still depends on cancer type (Zhang et al., 2020). From this IRG–TF regulatory network, we can see that in cervical cancer, STAT1 positively regulates low-risk IRGs (*PSME2*, *LTA*, and *PTPN6*), but STAT1 positively regulates high-risk IRGs (*OAS1*) in endometrial cancer, suggesting that transcription factor STAT1 may play different biological roles in these two types of cancers. The functional role of transcription factor FOXP3 is also unclear in existing studies. On the one hand, FOXP3 can act as a tumor suppressor in breast cancer (Zuo et al., 2007), ovarian cancer (Zhang and Sun, 2010), colon cancer (Li et al., 2013), and gastric cancer (Ma et al., 2013), but it can act as an oncogene in non-small cell lung cancer (Yang et al., 2017), lung adenocarcinoma (Li et al., 2016), and thyroid cancer (Chu et al., 2015). From this IRG–TF regulatory network, we can see that in cervical cancer, FOXP3 positively regulates low-risk IRGs (LTA and PTPN6) and positively regulates low-risk IRGs (LTA) in endometrial cancer, suggesting that FOXP3 may act as a tumor suppressor in these two types of cancers.

### Establishment and Evaluation of the IRGs Signature Model

**FIGURE 4:**
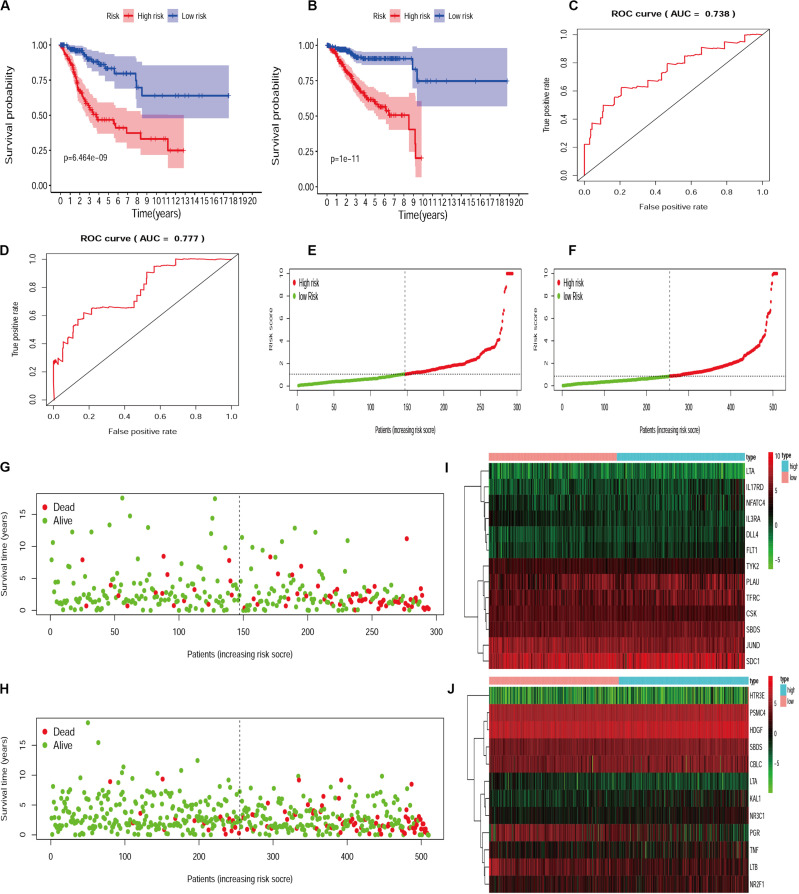
The evaluation of IRGs signature model in cervical cancer and endometrial cancer. Kaplan-Meier survival curves for cervical cancer **(A)** and endometrial cancer **(B)**. AUC for cervical cancer **(C)** and endometrial cancer **(D)**. The distribution and survival status of patients with risk score in the cervical cancer **(E,G)** and endometrial cancer **(F,H)**. The heatmap for IRGs in cervical cancer **(I)** and endometrial cancer **(J)**.

Since different IRGs expression profiles may indicate the differences in disease condition among patients, it is of significance to establish the IRGs’ prognosis signature model for patient risk evaluation. In this way, the IRGs’ prognosis signature models were established for cervical cancer and endometrial cancer, respectively (Supplementarys Table S1, S2). Patients were divided into a high-risk group and a low-risk group according to the median risk score. Calculation based on the IRGs prognosis signature model resulted in 147 and 147 patients assigned to high-risk and low-risk subsets, respectively, for those with cervical cancer, and similarly 255 and 255 patients to two subsets for those with endometrial cancer. Statistical evaluation was subsequently made to analyze this model by performing the comparison of Kaplan-Meier survival curves, evaluation of ROC curves, and drawing of distribution plots of patients at high/low risk. All these suggested that the IRGs signature model could be considered appropriate to evaluate the clinical prognosis of patients, with the exception of the moderately AUC (Figure 4).

### Multiple Evaluation of IRGs’ Signature Model Combined With Clinicopathology, Gene Expression Profiles, GSEA, and TIMER

**FIGURE 5:**
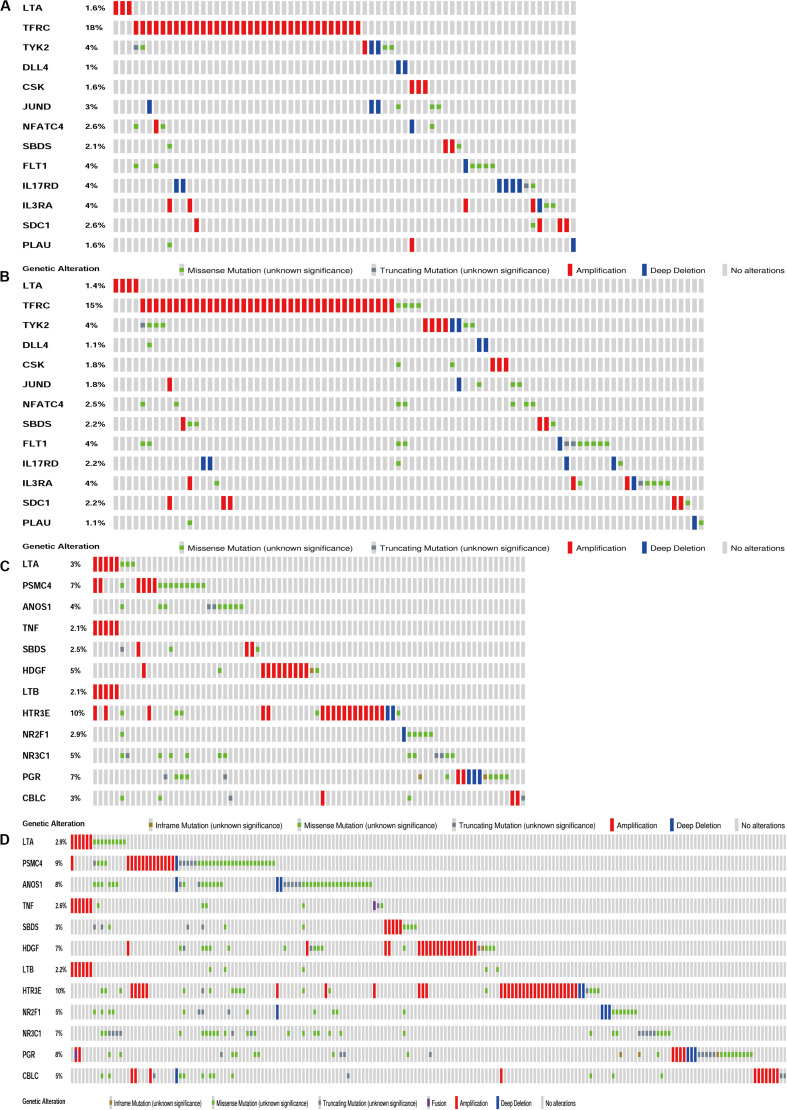
Genetic alteration landscape of IRGs in gene signatures model of CESC and UCEC. **(A)** Genetic alteration in the TCGA-CESC cohort (191 samples). **(B)** Genetic alteration in the TCGA-Pan Cancer Atlas cohort (278 samples). **(C)** Genetic alteration in the TCGA-UCEC cohort (242 samples). **(D)** Genetic alteration in the TCGA-Pan Cancer Atlas cohort (509 samples).

**FIGURE 6:**
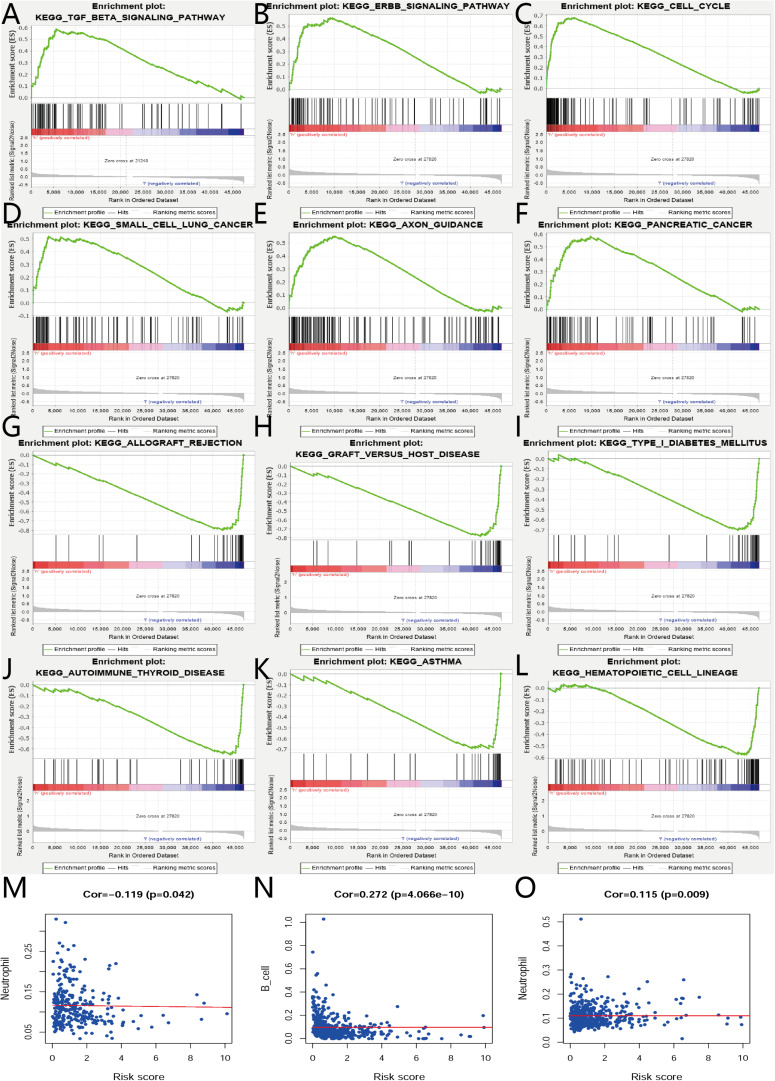
GSEA and relationships between IRGs and tumor infiltrating immune cells index in CESC and UCEC. **(A)** GSEA results of high risk group in cervical cancer. **(B–F)** GSEA results of high risk group in endometrial cancer. **(G–L)** GSEA results of low risk group in endometrial cancer. **(M)** Relationships between risk score of IRGs model in cervical cancer and tumor infiltrating immune cells index. **(N,O)** Relationships between risk score of IRGs model in endometrial cancer and tumor infiltrating immune cells index.

The biological characteristics regarding clinicopathology were also part of our considerations, including age, cancer stage, body mass index (BMI), and (TNM) stage, etc. We found the risk score resulted from the IRGs’ signature model could be satisfactory as an independent statistical measure to evaluate the risk levels of patients. As an exception, the IRGs signature model for endometrial cancer developed the following independent clinical measures for risk levels evaluation, including age, cancer stage, and tumor pathological grades. Statistical difference was observed in clinicopathological characteristics among many IRGs expression profiles. Based on the online database cbioportal, datasets of TCGA-CESC/TCGA-UCEC cohort and TCGA-PanCancer Atlas were applied (310 samples in CESC vs. 297 samples in PanCancer Atlas; 548 samples in UCEC vs. 529 samples in PanCancer Atlas). Only samples harboring both mutations and CAN data were included. In terms of CESC, IRGs were altered in 69 (36%) of 191 queried samples (TCGA-CESC) (Figure 5A), as compared with those altered IRGs detected in 88 (32%) of 278 queried samples (PanCancer Atlas) (Figure 5B). In terms of UCEC, IRGs were changed in 80 (33%) of 242 queried samples (TCGA-UCEC) (Figure 5C), compared with 192 (38%) of 509 samples (PanCancer Atlas) (Figure 5D). GSEA analysis in cervical cancer revealed that the high-risk group was significantly associated with the TGF-beta signaling pathway (NES = 2.059, FDR = 0.017) (Figure 6A), but no pathway was significantly relevant to the low risk group. However, the GSEA analysis in endometrial cancer showed that these pathways including erbb-signaling-pathway (NES = 2.099, FDR = 0.028), cell-cycle (NES = 2.195, FDR = 0.034), axon-guidance (NES = 2.106, FDR = 0.038), pancreatic-cancer (NES = 2.049, FDR = 0.039), and small-cell-lung-cancer (NES = 1.932, FDR = 0.048) were significantly relevant to the high risk group (Figures 6B–F), and graft-versus-host disease (NES = −1.916, FDR = 0.031), type-I-diabetes-mellitus (NES = −1.886, FDR = 0.034), allograft-rejection (NES = −1.989, FDR = 0.038), autoimmune-thyroid-disease (NES = −1.917, FDR = 0.038), hematopoietic-cell-lineage (NES = -1.831, FDR = 0.046), and asthma (NES = −1.928, FDR = 0.048) to the low risk group (Figures 6G–L). Finally, we evaluated the relationship between the IRG signature model and immune cell infiltration and thereby found that the infiltration of neutrophils was negatively correlated with the IRGs signature model for cervical cancer. However, the infiltration of B cells and neutrophils was positively correlated with this model for endometrial cancer (Figures 6M–O).

## Discussion

Many pre-existing scientific researches have demonstrated that the occurrence and progression of tumors are strongly related to immune cells and chemokines in the human body (Han et al., 2019; Rosenthal et al., 2019), which can be verified through the mechanism of immune escape (Luo et al., 2019). In some diseases and tumor biological processes, the changes of these immune biomarkers are clear and even can be predicted in the immune microenvironment (Silva-Santos et al., 2019). However, data on systematic and comprehensive molecular mechanisms in genome-wide profiling are limited for cervical cancer and endometrial cancer. Therefore, our study is designed to explore which types of IRGs show changes or may be going to change in patients with cervical cancer and endometrial cancer. Furthermore, we also investigated whether these differences could properly predict the clinical prognosis of patients and help to demonstrate the relationship between these IRGs and clinicopathological characteristics. This provides relevant information for us to develop better understanding on the biological changes of cervical and endometrial cancer.

Cancer cells have been shown to accumulate in inflammatory microenvironments, usually in the early stage of tumorigenesis (Hanahan and Weinberg, 2011); thus, it is genuinely helpful to identify differentially expressed IRGs in tumor tissues. Unfortunately, such studies on cervical cancer and endometrial cancer are rare. In this context, we extracted and calculated these differentially expressed IRGs for cervical cancer and endometrial cancer, respectively, resulting in a total of 146 differentially expressed IRGs that were shared by these two tumor types. Transcription factors (TFs), which also play a very important role in the human body, have been shown to regulate gene transcription at the nucleic acid level, thus affecting the expression of proteins (Lambert et al., 2018). After extraction and calculation of these differentially expressed TFs, 49 TFs were shared by these two diseases. Co-occurrence of differentially expressed IRGs and TFs is of great significance to guide our subsequent studies and those shared IRGs and TFs may suggest similar biological processes in both tumor types.

After that, enrichment analyses through GO and KEGG pathways were performed for these differentially expressed IRGs. The results showed genes were mainly enriched in the pathways including “cytokine-cytokine receptor interaction,” “Ras signaling pathway,” and “MAPK signaling pathway” for cervical cancer and the pathways including “cytokine-cytokine receptor interaction,” “chemokine signaling pathway,” and “PI3K-Akt signaling pathway” for endometrial cancer, indicating a possible relationship of these IRGs with tumor-associated development, progression, and invasion. Subsequently, we made further screening to select survival-associated IRGs. To investigate the relationship between these differentially expressed survival-associated IRGs and differentially expressed TFs, the regulatory network was constructed for these survival-associated IRGs and differentially expressed TFs, respectively. We found that the IRG LTA appeared in the regulatory network for both cancers. Previous studies have shown that the role of LTA varies with patient’s condition. In patients with breast cancer, LTA can be used as a possible tumor marker to evaluate the prognosis (Kohaar et al., 2009). In contrast, in the study of gastric cancer (Mou et al., 2015), the occurrence of gastric cancer is related to the genetic variation of LTA. However, no sufficient data is available to draw a comprehensive picture to characterize this gene, and additional studies are needed to make further demonstration.

In living organisms, biological processes are often characterized by involvement in various genes, multiple courses, and continuous biological responses; therefore, it is difficult to predict and explain condition changes and prognosis with a single or a small number of gene expression profiles. In this case, an overall analysis of “gene signatures” involving different genes provide us with a good method for predicting the prognosis of patients. As early as in 2007, scholars (Chen et al., 2007) used a gene signature model to evaluate the clinical prognosis of non-small cell lung cancer (NSCLC), and the results demonstrated high reliability of this method. Since then, this kind of multigene signature model has been applied more frequently in other diseases, for example, ovary cancer (An et al., 2018), lung cancer (Liu et al., 2019a), colon cancer (Mo et al., 2019), lung adenocarcinoma (Wang et al., 2019), and colorectal cancer (Zhou et al., 2019). Based on these findings, we decided to establish an IRGs signature model for cervical cancer and endometrial cancer to evaluate the prognosis of patients.

For the establishment of gene signature models, 25/23 survival-associated IRGs were selected for cervical cancer and endometrial cancer, respectively. As a result, 13 IRGs in cervical cancer and 12 IRGs in endometrial cancer were found appropriate to establish the model. We used the two IRGs signature models to calculate the risk levels for each patient and found the differences in Kaplan-Meier survival curves were statistically significant between high- and low-risk groups for both cervical cancer (*p* = 6.464e-9) and endometrial cancer (*p* = 1e-11). In terms of survival and death, statistics of patients were also significantly different between high- and low-risk groups. These data obtained from evaluation models suggested that the IRGs’ signature model may be a good way to assess the risk levels of patients; however, the area under the ROC curve (AUC) moderately only reached to about 0.738 in cervical cancer and 0.777 in endometrial cancer.

**FIGURE 7:**
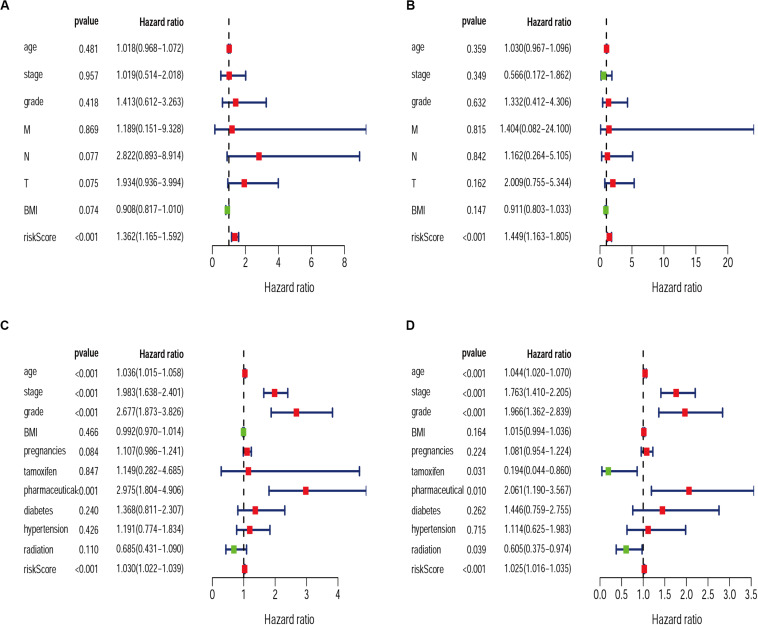
Univariate and Multivariate Cox regression analysis of clinical characteristics for CESC **(A,B)** and UCEC **(C,D)**.

Exploring the relationship between clinicopathological characteristic and patient prognosis can provide us with more valuable information. Based on univariate and multivariate Cox regression analyses, we found the risk score resulted from the IRGs signature model could be considered an independent statistical measure to evaluate the overall survival (OS) in patients with cervical cancer (*P* < 0.001) (Figures 7A,B). Similar findings were obtained for the risk score resulted from the IRG signature model for endometrial cancer (Figures 7C,D), where independent clinical measures included age (*P* < 0.001), cancer stage (*P* < 0.001), tumor pathological grade (*P* < 0.001), the use of estrogen antagonist tamoxifen (Gaber-Wagener and Marth, 2020) (*P* < 0.05), and radiation therapy (Mirza, 2020) (*P* < 0.05). These results were highly consistent with previous studies (Casablanca et al., 2019; Trojano et al., 2019; Wu et al., 2019).

**FIGURE 8:**
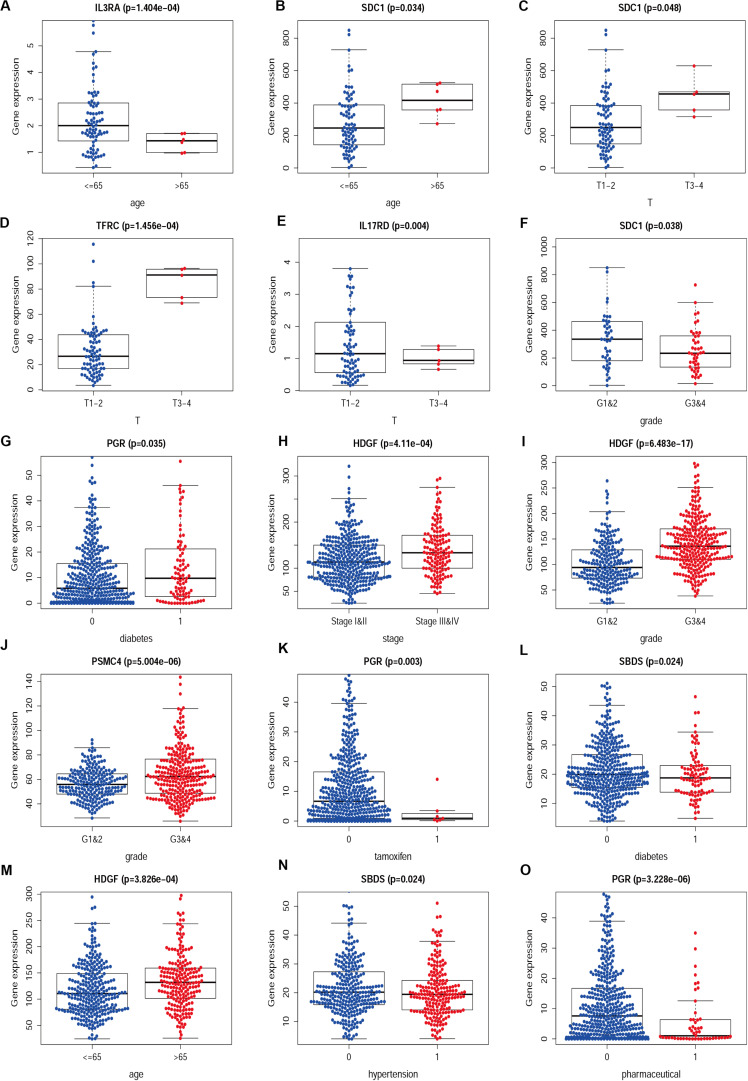
The difference between IRGs expression profile and clinicopathological characteristics in CESC **(A–F)** and UCEC **(G–O)**. Show part only.

We then examined the correlation between the risk score and clinicopathological characteristics of the patients. However, there was no statistical difference in either the risk score proved by the IRGs’ signature model or the clinicopathological characteristics between patients with cervical cancer and those with endometrial cancer, but statistical difference was observed in the IRG expression profiles and clinicopathological characteristics (Tables 1, 2, Figure 8 and Supplementary Table S3) for which further investigation could be considered. GSEA analysis for cervical cancer indicated that the high-risk group was significantly associated with the TGF beta signaling pathway, while in endometrial cancer the results showed relevance to erbb signaling pathway, cell cycle, axon guidance pathway, pancreatic cancer, and small-cell lung cancer. These pathways were associated with tumor development and progression, suggesting that these molecular pathways were likely to be activated in high-risk groups. Thus, the validity of this IRGs signature model for predicting risk scores was well established.

**TABLE 1:** Relationships between the expressions of the IRGs and the clinicopathological characteristics in cervical cancer.

Id	Age	Stage	Grade	M	N	T	BMI
	t(p)	t(p)	t(p)	t(p)	t(p)	t(p)	t(p)
LTA	−1.168(0.293)	0.527 (0.608)	−1.462(0.148)	1.984 (0.103)	0.07 (0.945)	0.668 (0.529)	−2.633(0.010)
TFRC	−0.149(0.886)	0.811 (0.431)	1.066 (0.290)	1.004 (0.364)	−1.127(0.271)	−7.596(1.456*e*−04)	0.948 (0.347)
TYK2	0.31 (0.766)	−0.047(0.963)	−0.252(0.802)	−0.764(0.500)	0.503 (0.618)	1.611 (0.162)	−0.068(0.946)
DLL4	1.257 (0.235)	−0.259(0.798)	0.038 (0.970)	−0.065(0.952)	0.846 (0.403)	−0.818(0.452)	0.249 (0.804)
CSK	0 (1.000)	−1.261(0.224)	−0.23(0.819)	1.887 (0.138)	0.102 (0.919)	2.566 (0.050)	1.782 (0.081)
JUND	0.721 (0.492)	1.523 (0.153)	0.764 (0.448)	−0.819(0.472)	−0.067(0.947)	−0.473(0.659)	−1.485(0.142)
NFATC4	1.557 (0.156)	0.66 (0.520)	0.178 (0.859)	−0.846(0.458)	−0.016(0.987)	0.933 (0.396)	−1.993(0.050)
SBDS	−0.446(0.669)	−0.019(0.985)	0.643 (0.522)	0.884 (0.438)	−0.667(0.511)	−0.676(0.535)	1.376 (0.176)
FLT1	0.348 (0.736)	−0.347(0.733)	−0.478(0.634)	0.6 (0.588)	−0.068(0.946)	−1.14(0.298)	−0.061(0.952)
IL17RD	0.521 (0.617)	−0.709(0.490)	−0.475(0.636)	0.46 (0.670)	0.734 (0.467)	3.091 (0.004)	−2.338(0.022)
IL3RA	4.431(1.404*e*−04)	0.905 (0.378)	−1.234(0.221)	0.094 (0.930)	−0.918(0.365)	1.262 (0.261)	−2.366(0.020)
SDC1	−2.491(0.034)	0.228 (0.823)	2.122 (0.038)	1.035 (0.370)	−1.724(0.098)	−2.498(0.048)	1.131 (0.261)
PLAU	−0.922(0.394)	−1.368(0.189)	−0.344(0.732)	1.174 (0.314)	0.517 (0.609)	−0.892(0.416)	2.693 (0.010)
riskScore	−1.076(0.312)	0.154 (0.880)	1.254 (0.215)	1.864 (0.103)	−0.695(0.495)	−1.819(0.138)	0.182 (0.856)

**t, t-value of student’s t-test; P, P-value of student’s t-test.**

**TABLE 2:** Relationships between the expressions of the IRGs and the clinicopathological characteristics in endometrial cancer.

Id	Age	Stage	Grade	BMI	Pregnancies	Tamoxifen	Pharmaceutical	Diabetes	Hypertension	Radiation
	t(p)	t(p)	t(p)	t(p)	t(p)	t(p)	t(p)	t(p)	t(p)	t(p)
LTA	0.908 (0.364)	2.775 (0.006)	−0.665 (0.507)	0.584 (0.561)	0.051 (0.959)	−0.384 (0.714)	0.354 (0.725)	0.351 (0.726)	−0.705 (0.481)	−0.26 (0.795)
PSMC4	−1.329 (0.184)	−2.344 (0.020)	−4.647 (5.004e−06)	−0.321 (0.749)	−1.626 (0.106)	−0.153 (0.882)	0.062 (0.951)	0.022 (0.982)	−1.175 (0.241)	−1.446 (0.150)
KAL1	−2.104 (0.036)	−1.024 (0.307)	−1.559 (0.120)	−2.168 (0.032)	−0.839 (0.403)	−1.101 (0.313)	−0.397 (0.693)	−0.536 (0.593)	0.088 (0.930)	−0.509 (0.611)
TNF	−2.04 (0.042)	−1.555 (0.122)	−1.91 (0.057)	0.44 (0.661)	−0.712 (0.477)	−0.886 (0.410)	−1.937 (0.058)	−1.535 (0.127)	−0.468 (0.640)	0.647 (0.518)
SBDS	−0.653 (0.514)	−3.11 (0.002)	−7.062 (6.468e−12)	1.414 (0.162)	−0.376 (0.707)	1.142 (0.294)	−0.28 (0.780)	2.266 (0.024)	2.271 (0.024)	−1.578 (0.115)
HDGF	−3.582 (3.826e−04)	−3.583 (4.11e−04)	−8.674 (6.483e−17)	1.154 (0.251)	−1.512 (0.132)	−0.69 (0.515)	−1.805 (0.076)	1.174 (0.242)	0.528 (0.597)	−1.28 (0.201)
LTB	−1.397 (0.163)	−0.288 (0.773)	−0.715 (0.475)	0.823 (0.413)	0.72 (0.472)	−0.882 (0.411)	−0.218 (0.828)	0.306 (0.760)	0.951 (0.342)	1.553 (0.121)
HTR3E	0.9 (0.369)	−1.025 (0.307)	−1.06 (0.290)	−1.06 (0.290)	1.053 (0.293)	0.511 (0.611)	0.873 (0.383)	1.017 (0.310)	0.814 (0.416)	−0.98 (0.328)
NR2F1	1.173 (0.242)	0.118 (0.906)	−1.776 (0.077)	0.858 (0.393)	0.953 (0.341)	0.511 (0.626)	1.934 (0.056)	0.24 (0.811)	0.876 (0.382)	0.023 (0.982)
NR3C1	−1.703 (0.090)	−3.071 (0.002)	−5.572 (4.443e−08)	1.248 (0.216)	−0.612 (0.541)	−1.169 (0.286)	−1.606 (0.113)	−0.234 (0.815)	0.705 (0.481)	−1.204 (0.230)
PGR	4.139 (4.121e−05)	5.709 (2.527e−08)	8.551 (3.876e−16)	−4.745 (5.258e−06)	0.449 (0.654)	4.321 (0.003)	4.974 (3.228e−06)	−2.129 (0.035)	−1.698 (0.090)	0.865 (0.387)
CBLC	−1.752 (0.081)	−1.01 (0.314)	−1.915 (0.056)	−0.431 (0.667)	−0.266 (0.790)	−0.331 (0.750)	−0.139 (0.890)	0.031 (0.976)	1.097 (0.273)	0.107 (0.915)
riskScore	1 (0.318)	−1 (0.319)	−1 (0.318)	−1 (0.318)	1 (0.318)	1 (0.318)	1 (0.318)	1 (0.318)	1 (0.318)	−1 (0.319)

**t, t-value of student’s t-test; P, P−value of student’s t-test.**

By evaluating the relationship between the risk score provided by the IRGs’ signature model and immune cell infiltration, we found that neutrophil infiltration was negatively correlated with risk scores in cervical cancer; however, the infiltration of immune B cells and neutrophils were positively correlated with risk scores in endometrial cancer. Studies (Dong et al., 2019) have shown that the ratio of neutrophils to lymphocytes is an independent measure to determine prognosis and lymph node metastasis in endometrial cancer (Aoyama et al., 2019). It is also reported that (Wisdom et al., 2019) neutrophils can increase the resistance of tumor cells to radiation therapy, and neutrophil-lymphocyte ratio can be considered as a predictor in stage IVB or recurrent cervical cancer patients treated by chemotherapy. Neutrophil-lymphocyte ratio ≥ 3.6 has been identified as an independent predictor of poor oncologic outcomes with respect to OS (Farzaneh et al., 2019; Ittiamornlert and Ruengkhachorn, 2019). These data suggest that the relationship between the risk score provided by the IRG signature model and neutrophil infiltration is well-established. The determination for B lymphocytes still requires data due to lack of relevant studies on cervical and endometrial cancers.

## Conclusion

In conclusion, our analyses for this IRG signature model still leave some limitations for us to improve. First of all, in cervical and endometrial cancer, there are different pathological types, but no different pathological type models have been developed yet. As such, there might be differences for some special pathological types. Second, we just selected immune-related genes to establish the IRG signature model, whereas in the human body, the occurrence and progression of cancer or other diseases is a comprehensive process that involves nucleic acid transcriptome, proteomics, minerals, and other important elements. As the present study focuses on transcriptome of RNA, there may exist a certain selection bias in this model. Third, our model lacks independent databases for verification which is why we are very careful to draw these conclusions. At last, these models are provided with *in vivo* and *in vitro* experimental data. Although the model developed by us has some shortages, we still hope to provide new ideas and guidance for future researches in the treatment of cervical cancer and endometrial cancer.

## Data Availability Statement

Publicly available datasets were analyzed in this study. This data can be found here: https://portal.gdc.cancer.gov.

## Author Contributions

HD and G-LF contributed to the conception of the study. Y-XY contributed significantly to analysis and manuscript preparation. HD, X-XX, and OM performed the data analyses and wrote the manuscript. WZ helped perform the analysis with constructive discussions. Final approval of manuscript by all authors.

## Conflict of Interest

The authors declare that the research was conducted in the absence of any commercial or financial relationships that could be construed as a potential conflict of interest.

## Footnotes

1 http://www.webgestalt.org/

2 http://www.Cistrome.org/

3 https://www.cbioportal.org/

4 https://cistrome.shinyapps.io/timer/

## Abbreviations

AUC: An area under the ROC curve
FDR: false discovery rate
GO: gene ontology
GSEA: Gene Set Enrichment Analysis
HPV: human papillomavirus
IRGs: immune-related genes
KEGG: Kyoto Encyclopedia of Genes and Genomes
OS: overall survival
ROC: Receiver Operating Characteristic curve
TCGA: The Cancer Genome Atlas
TFs: transcription factors
TIMER: Tumor IMmune Estimation Resource.
