# Supplementary material for: Prognostic Implications of Immune-Related Genes’ (IRGs) Signature Models in Cervical Cancer and Endometrial Cancer
Source: Front Genet. 2020 Jul 21;11:725. doi: 10.3389/fgene.2020.00725 (PMC7385326; doi:10.3389/fgene.2020.00725)
Supplement: Supplementary file 1 [file Data_Sheet_1.docx]

**[Supplementary](javascript:;) Material**

**[Supplementary](javascript:;) Table S1**. The formula for the signature model of cervical cancer (CESC) and endometrial cancer (UCEC).

| The formula for cervical cancer was shown as follows: |
| --- |
| [Expression level of LTA * (0.477)] + [Expression level of TFRC * (0.005)] + [Expression level of TYK2 * (-0.040)] + [Expression level of DLL4 * (0.488)] + [Expression level of CSK * (-0.042)] + [Expression level of JUND * (-0.003)] + [Expression level of NFATC4 * (0.124)] + [Expression level of SBDS* (0.011)] + [Expression level of FLT1* (-0.161)] + [Expression level of IL17RD *(0.126)] + [Expression level of IL3RA * (-0.408)] + [Expression level of SDC1* (0.001) + [Expression level of PLAU * (0.002)] |
| The formula for endometrial cancer was shown as follows: |
| [Expression level of LTA * (-0.813)] + [Expression level of PSMC4 * (0.002)] + [Expression level of KAL1 * (0.118)] + [Expression level of TNF * (0.041)] + [Expression level of SBDS* (0.015)] + [Expression level of HDGF * (0.002)] + [Expression level of LTB *( -0.03)] + [Expression level of HTR3E * (0.605)] + [Expression level of NR2F1 * (0.015) + [Expression level of NR3C1 * (0.304)] + [Expression level of PGR * (-0.026)] + [Expression level of CBLC * (0.012)] |

[**Supplementary**](javascript:;)**Table S2**. General characteristics of immune‐related genes(IRGs) in cervical cancer (CESC) and endometrial cancer (UCEC).

| Genes  (CESC) | LogFC | HR | HR.95L | HR.95H | pvalue |
| --- | --- | --- | --- | --- | --- |
| LTA | 3.1806 | 1.61270239 | 0.866373232 | 3.001949855 | 0.131681462 |
| TFRC | 2.34911 | 1.005911338 | 1.0022098 | 1.009626548 | 0.001727313 |
| TYK2 | 1.16629 | 0.960252524 | 0.911281266 | 1.011855444 | 0.12884646 |
| DLL4 | -1.9798 | 1.629934032 | 1.18514298 | 2.241657754 | 0.0026586 |
| CSK | 1.23511 | 0.958583033 | 0.93207351 | 0.985846526 | 0.003114716 |
| JUND | -1.509 | 0.99697404 | 0.992677318 | 1.00128936 | 0.169057251 |
| NFATC4 | -2.583 | 1.132522897 | 1.026193647 | 1.249869472 | 0.013361631 |
| SBDS | -1.0128 | 1.012010985 | 1.005846144 | 1.018213611 | 0.000128282 |
| FLT1 | -2.0832 | 0.851147734 | 0.681264372 | 1.063394028 | 0.155942113 |
| IL17RD | -2.7483 | 1.134526551 | 1.040597332 | 1.236934263 | 0.004203294 |
| IL3RA | -1.8112 | 0.664652312 | 0.500026234 | 0.883479038 | 0.004906105 |
| SDC1 | 4.81268 | 1.001018635 | 1.000106444 | 1.001931659 | 0.028612127 |

Note: FC: fold change; HR: hazard ratio

| Genes  (UCEC) | LogFC | HR | HR.95L | HR.95H | pvalue |
| --- | --- | --- | --- | --- | --- |
| LTA | 1.49205 | 0.443164013 | 0.212415507 | 0.924576293 | 0.0300852 |
| PSMC4 | 1.04783 | 1.002525813 | 0.999903369 | 1.005155135 | 0.059072587 |
| KAL1 | 1.65627 | 1.125330003 | 1.051827039 | 1.203969444 | 0.000612302 |
| TNF | 2.28677 | 1.042179916 | 1.020386975 | 1.0644383 | 0.000127233 |
| SBDS | -1.2653 | 1.015604893 | 1.003226282 | 1.028136241 | 0.013332207 |
| HDGF | 1.21742 | 1.002956971 | 0.999246796 | 1.006680921 | 0.118409826 |
| LTB | 2.29615 | 0.970292572 | 0.948226327 | 0.992872322 | 0.010187111 |
| HTR3E | 3.34899 | 1.832263789 | 1.512046779 | 2.220295456 | 6.46E-10 |
| NR2F1 | -2.3036 | 1.015913419 | 1.001476595 | 1.030558357 | 0.030616707 |
| NR3C1 | -2.4084 | 1.355469829 | 1.124580973 | 1.63376271 | 0.001411529 |
| PGR | -1.5942 | 0.973747448 | 0.948289756 | 0.999888576 | 0.049044108 |
| CBLC | 2.36437 | 1.012448934 | 1.006572116 | 1.018360064 | 3.11E-05 |

Note: FC: fold change; HR: hazard ratio

| Cases(CESC) |  | Group | Number |
| --- | --- | --- | --- |
| Age | Normal | / | |
|  | Tumor | <=65 | 270 |
|  |  | >65 | 34 |
| Stage | Normal | / | |
|  | Tumor | Stage I | 164 |
|  |  | Stage II | 67 |
|  |  | Stage III | 45 |
|  |  | Stage IV | 22 |
|  |  | unkonwn | 7 |
| Grade | Normal | / | |
|  | Tumor | G1 | 22 |
|  |  | G2 | 133 |
|  |  | G3 | 122 |
|  |  | G4 | 1 |
|  |  | GX | 27 |
| BMI | Normal | / | |
|  | Tumor | <25 | 99 |
|  |  | >=25 | 163 |
|  |  | unkonwn | 42 |
| M | Normal | / | |
|  | Tumor | M0 | 109 |
|  |  | M1 | 11 |
|  |  | MX | 184 |
| N | Normal | / | |
|  | Tumor | N0 | 135 |
|  |  | N1 | 60 |
|  |  | NX | 109 |
| T | Normal | / | |
|  | Tumor | T1 | 143 |
|  |  | T2 | 71 |
|  |  | T3 | 19 |
|  |  | T4 | 10 |
|  |  | TX | 61 |

**[Supplementary](javascript:;) Table S3**. Characteristics of TCGA cervical cancer (CESC) and endometrial cancer (UCEC) cohorts.

Note: GX, TX, MX, NX and unkonwn represent unavailable or unclear data.

| Cases(UCEC) |  | Group | Number |
| --- | --- | --- | --- |
| Age | Normal | / | |
|  | Tumor | <=65 | 305 |
|  |  | >65 | 135 |
|  |  | unkonwn | 3 |
| Stage | Normal | / | |
|  | Tumor | Stage I | 339 |
|  |  | Stage II | 51 |
|  |  | Stage III | 124 |
|  |  | Stage IV | 29 |
| Grade | Normal | / | |
|  | Tumor | G1 | 98 |
|  |  | G2 | 120 |
|  |  | G3 | 314 |
|  |  | G4 | 11 |
| BMI | Normal | / | |
|  | Tumor | <25 | 95 |
|  |  | >=25 | 417 |
|  |  | unkonwn | 31 |
| Pregnancies | Normal | / | |
|  | Tumor | 0 | 65 |
|  |  | 1 | 51 |
|  |  | 2 | 114 |
|  |  | 3 | 66 |
|  |  | 4+ | 74 |
|  |  | unkonwn | 173 |
| Tamoxifen | Normal | / | |
|  | Tumor | YES | 41 |
|  |  | NO | 245 |
|  |  | unkonwn | 257 |
| Pharmaceutical | Normal | / | |
|  | Tumor | YES | 28 |
|  |  | NO | 13 |
|  |  | unkonwn | 502 |
| Diabetes | Normal | / | |
|  | Tumor | YES | 99 |
|  |  | NO | 265 |
|  |  | unkonwn | 179 |
| Hypertension | Normal |  |  |
|  | Tumor | YES | 228 |
|  |  | NO | 162 |
|  |  | unkonwn | 153 |
| Radiation | Normal |  |  |
|  | Tumor | YES | 223 |
|  |  | NO | 284 |
|  |  | unkonwn | 36 |

Note: The unkonwn represent unavailable or unclear data.
